# The Mutual Relationship between Glycosylation and Non-Coding RNAs in Cancer and Other Physio-Pathological Conditions

**DOI:** 10.3390/ijms232415804

**Published:** 2022-12-13

**Authors:** Martina Duca, Nadia Malagolini, Fabio Dall’Olio

**Affiliations:** Department of Experimental, Diagnostic and Specialty Medicine (DIMES), General Pathology Building, University of Bologna, Via San Giacomo 14, 40126 Bologna, Italy

**Keywords:** glycosylation, glycosyltransferases, non-coding RNAs, miRNA, sugar antigens

## Abstract

Glycosylation, which consists of the enzymatic addition of sugars to proteins and lipids, is one of the most important post-co-synthetic modifications of these molecules, profoundly affecting their activity. Although the presence of carbohydrate chains is crucial for fine-tuning the interactions between cells and molecules, glycosylation is an intrinsically stochastic process regulated by the relative abundance of biosynthetic (glycosyltransferases) and catabolic (glycosidases) enzymes, as well as sugar carriers and other molecules. Non-coding RNAs, which include microRNAs, long non-coding RNAs and circRNAs, establish a complex network of reciprocally interacting molecules whose final goal is the regulation of mRNA expression. Likewise, these interactions are stochastically regulated by ncRNA abundance. Thus, while protein sequence is deterministically dictated by the DNA/RNA/protein axis, protein abundance and activity are regulated by two stochastic processes acting, respectively, before and after the biosynthesis of the protein axis. Consequently, the worlds of glycosylation and ncRNA are closely interconnected and mutually interacting. In this paper, we will extensively review the many faces of the ncRNA–glycosylation interplay in cancer and other physio-pathological conditions.

## 1. Introduction

Glycosylation consists of the enzymatic addition of sugars or sugar chains to proteins or lipids, giving rise to glycoproteins and glycolipids, respectively. Glycans are attached to proteins usually through an amidic linkage to asparagine (*N*-linked) or to the hydroxyl group of serine or threonine (*O*-linked). The presence of these sugar chains exerts a subtle but crucial functional effect, modulating the interactions between molecules and cells. Glycans are deeply altered in pathological conditions, including cancer [1], inflammation [2] and aging [3,4]. Glycan structure is not under direct genetic control, but results from the cooperative and competitive interaction between glycosyltransferases. These enzymes transfer a monosaccharide from an activated sugar donor (frequently a nucleotide-sugar) to an acceptor, which can be an amino acid, a lipid or another sugar. Consequently, glycosylation can be considered a stochastic rather than a deterministic process. The biological role of glycans is frequently mediated by sugar-binding molecules (lectins) which, upon recognition of specific carbohydrate structures, trigger a broad range of cellular effects, including proliferation, apoptosis and cell migration. Galectins and siglecs are among these sugar-binding molecules. The extraordinary importance of non-coding RNAs (ncRNAs)—which include micro RNAs (miRNAs), long non-coding RNAs (lncRNAs) and circular RNAs (circRNAs)—in gene expression regulation, both at the transcriptional and the post-transcriptional level, is increasingly being recognized. Together, ncRNAs form an extremely complex non-deterministic network of gene expression regulation. Many glycosyltransferases and sugar-binding molecules have been shown to be regulated by ncRNAs. On the other hand, glycosylation has been demonstrated to modulate ncRNA expression in some instances. Finally, small ncRNAs have recently been shown to undergo canonical *N*-glycosylation [5]. These glyco-RNAs bear terminal sialic acid (Sia) and fucose (Fuc) residues and can interact with sugar-binding molecules, such as siglecs [5]. Thus, ncRNA network and glycosylation can be considered as two stochastic mechanisms affecting the role of protein: the first acting before, and the second after, protein axis biosynthesis. The purpose of this review is to provide an overview of the emerging picture of the glycans–ncRNA bi-directional relationship.

## 2. The Essentials of Non-Coding RNAs

RNA-seq technologies revealed that while the human genome is widely transcribed, only a small percentage of RNA (~2%) is protein-coding, broadening the spectrum of RNAs involved in gene expression regulation. According to their length, ncRNAs can be grouped in two main classes: miRNAs and lncRNAs [6].

### 2.1. miRNA

Mature miRNAs are 22-nucleotide (nt)-long RNAs. They are transcribed by RNA polymerase II into a primary form called (pri)-miRNA and undergo a maturation process, first in the nucleus (cropping), then in the cytosol (dicing), by the ribonucleases Drosha and Dicer, respectively. In the nucleus, (pri)-miRNA are converted by Drosha into miRNA precursors, then translocated in the cytosol where they are cleaved by Dicer into double-stranded mature miRNA. Only the guide strand from the miRNA duplex is incorporated in RNA-induced silencing complex (RISC), and it directs the RISC to degrade the complementary target-mRNA. Although post-transcriptional gene regulation represents the main function of miRNAs, they also exert control over other ncRNAs, interacting with lncRNAs and circRNAs [7]. Typically, both the gene locus and precursor miRNA (pre-miRNA) of a miRNA is referred as “mir”, while the mature miRNA product is designated “miR”. miRNA are defined by the prefix miR, followed by a number (e.g., miR-515). A three-letter code prefix defines the species (e.g., hsa-mir-515 indicates Homo sapiens). This can be followed by a letter (e. g. miR-515a and miR-515b) if the miRNA with the same number diverge for one or two nucleotides. These miRs belong to the same family. If two diverse loci produce identical mature products, an additional number is given after the full name. For instance, mir-515-1 and mir-515-2 produce the same final miRNA product: miR-515. To indicate whether the mature sequence comes from the 5’ arm or the 3’ arm of the precursor, the -5p or the -3p suffix are added (e.g., miR-515-5p or miR-515-3p).

### 2.2. lncRNAs

**Figure 1:**
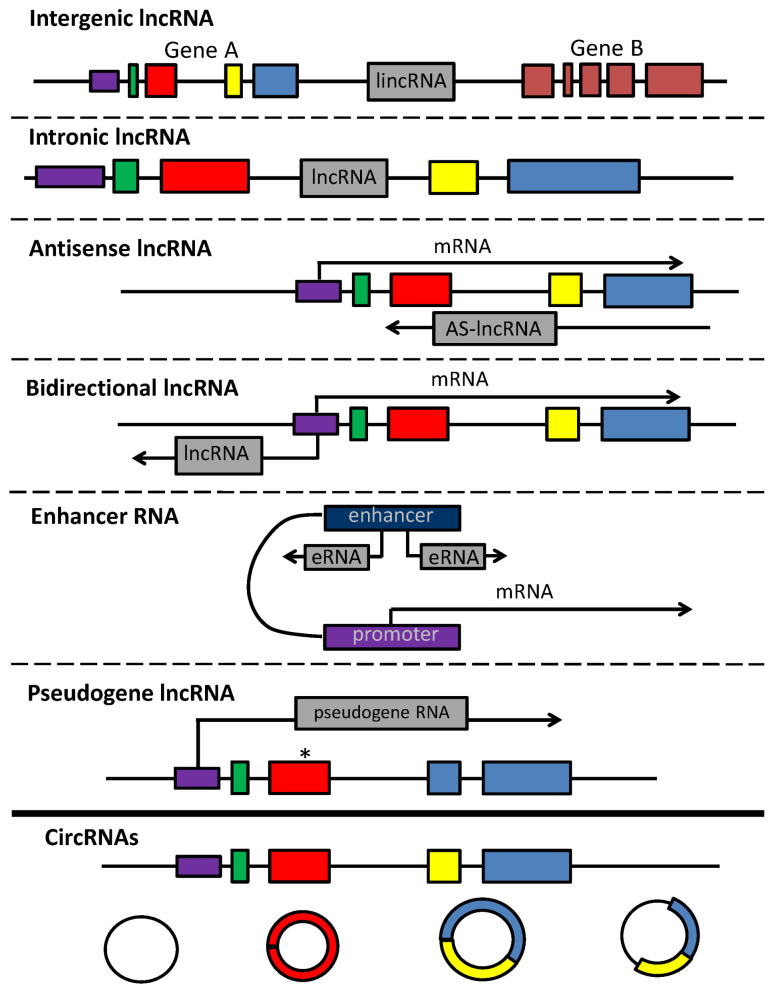
LncRNAs and circRNAs. Gene A comprises 4 exons (in green, red, yellow and blue, respectively). Its promoter is depicted in violet. Intergenic lncRNAs are generated by transcription of sequences between genes; intronic lncRNAs are generated by transcription of intronic regions between exons of a coding gene; antisense lncRNAs are produced by transcription of the antisense DNA strand; bidirectional lncRNAs are produced from the antisense transcription starting from the promoter of the coding gene; enhancer RNAs are derived from enhancer sequences; pseudogene lncRNAs are produced by transcription of genes carrying inactivating mutations (pseudogenes). Mutation is marked by an asterisk. circRNAs are produced from (left to right) intronic sequences, single exons, multiple exons and intronic and exonic sequences.

LncRNAs are >200-nt-long transcripts that do not encode for proteins. However, lncRNAs share common traits with mRNAs, since most of them are transcribed by RNA polymerase II and undergo specific post-transcriptional modifications, such as capping, polyadenylation and alternative splicing [8]. Depending on their position in the genome, lncRNAs are referred to as “intergenic” (lincRNAs) when localized between two protein-coding genes (Figure 1); “intronic” (lncRNA) when situated within the intronic portion of a protein-coding gene; “antisense” (AS-lncRNAs) when derived from the antisense RNA strand of protein-coding gene; “bidirectional” when transcribed from the same promoter of protein-coding gene, but in the opposite direction; and enhancer RNAs (eRNAs) when derived from enhancer regions, aiding the transcription factor placement in the proximity of promoters. LncRNAs are also generated by transcription of pseudogenes (genes carrying one or more mutations affecting their RNA translation). Other than linear lncRNAs, circRNAs represent the most abundant isoform, resulting from both canonical and back-splicing [6]. Due to their strong tissue-specific expression, lncRNAs exert a key role in several physiological processes such as cell cycle, differentiation and metabolism, and their dysregulation may lead to disease, such as cancer and infections. LncRNA display different mechanisms of action depending on their localization in the nucleus or cytoplasm [8]. In the nucleus, they can modulate transcription through the recruitment of TF to the promoter (eRNAs) or through the direct interaction with the RNA pol II (circRNAs). LncRNAs can also induce epigenetic modifications via histone remodeling. In the cytoplasm, lncRNAs act post-transcriptionally by interacting with RNAs and/or proteins. For instance, lncRNAs regulate alternative splicing, mRNA stability and RNA availability. Both linear and circular lncRNAs can act as molecular sponges by harboring a genomic region complementary to miRNA that competes with miRNA for the target site of mRNA (competitive endogenous RNAs, ceRNA). lncRNAs sequester miRNA, thus impairing the interaction with miRNA-target RNA and preventing targeted mRNA degradation [6].

## 3. The Essentials of Glycosylation

### 3.1. N-Glycosylation

**Figure 2:**
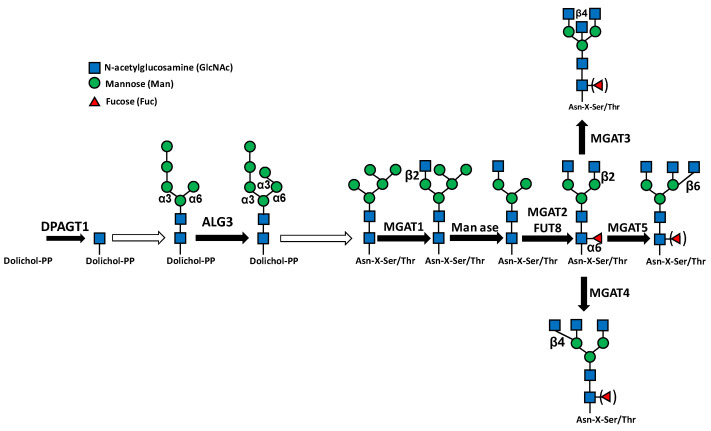
*N*-glycosylation steps regulated by ncRNAs. Black arrows indicate single-step reactions. White arrows indicate that the indicated transition is the product of multiple steps.

*N*-glycosylation starts in the rough endoplasmic reticulum (RER) with the building of an oligosaccharide comprising three *N*-acetylglucosamine (GlcNAc), nine mannose (Man) and three glucose (Glc) residues on the dolichol phosphate lipid-carrier [9,10] (Figure 2). This “high mannose” molecule is subsequently transferred “en bloc” to an Asn-X-Ser/Thr consensus motif of a nascent polypeptide chain. After transfer to protein, the high-mannose oligosaccharide undergoes sequential trimming of the three Glc residues and of six Man residues, followed by the building and elongation of the outer branches by the addition of GlcNAc and Gal residues. At these stages, the addition of a “core fucose” to the innermost GlcNAc residue can also occur. Finally, these branches can be elongated and capped, usually by Sia and/or Fuc. Sialic acid is always in the terminal position of the branches and can be followed only by successive Sia residues. 

### 3.2. O-Glycosylation

**Figure 3:**
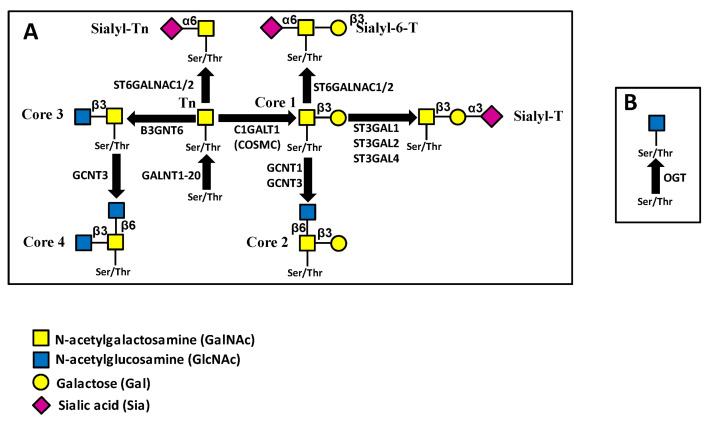
Pathways of *O*-glycosylation. (**A**) mucin-type; (**B**) *O*-GlcNAc. Enzymes catalyzing the same reactions but not mentioned in the text are not shown.

*O*-glycosylation consists of the stepwise addition of single sugars to the oligosaccharide chains of glycoproteins during their transit along the exocytic pathway [11]. In the canonical “mucin-type” *O*-glycosylation, linkage with peptide involves the *N*-acetylgalactosamine (GalNAc) terminal residue (Figure 3A). This step can be catalyzed by 20 different GalNAc transferases, with subtle differences in substrate specificities [12]. Subsequently, a defined number of basic “core” structures are synthesized, elongated and finally “capped”, usually by Sia and Fuc (Figure 3A). A peculiar type of glycosylation, represented by the addition of a single GlcNAc residue to Ser/Thr (*O*-GlcNAcylation), is mediated by the *O*-GlcNAc transferase product of the *OGT* gene [13] (Figure 3B). Unlike conventional glycosylation, *O*-GlcNAcylation regards cytoplasmic and nuclear proteins and competes with phosphorylation for the post-translational modification of Ser/Thr residues. OGT plays a particularly relevant role in regulating gene expression because GlcNAcylation of Polycomb group proteins is necessary for their transcriptional repression activity [14,15].

### 3.3. Glycolipids

**Figure 4:**
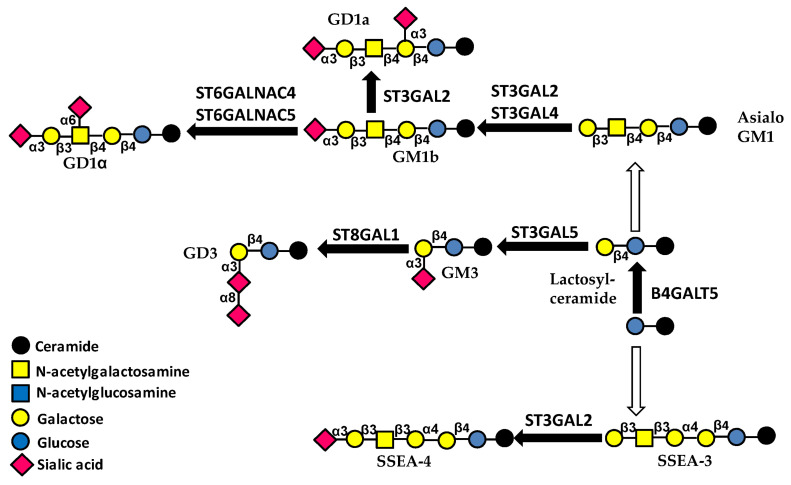
Pathways of glycolipid biosynthesis. Black arrows indicate single-step reactions. White arrows indicate that the indicated transition is the product of multiple steps. Enzymes catalyzing the same reactions but not mentioned in the text are not shown.

Glycolipid biosynthesis starts with the addition of glucose (Glc) to the lipid ceramide, followed by the addition of galactose by B4GALT5 (Figure 4). To this core structure, a Sia residue can be added by sialyltransferase ST3GAL5 forming GM3 ganglioside. Gangliosides are sialylated glycolipids, and GM3 is the simplest member of this category. 

## 4. Regulation of Glycosylation by ncRNAs

Non-coding RNAs regulate multiple steps of the biosynthesis of *N*- and *O*-glycans. In a pioneering work, Kurcon et al. [16] used miR of the 200 family (miR-200f), known to regulate the epithelial to mesenchymal transition (EMT), as a proxy to identify glycosyltransferases involved in EMT. ST3GAL5 and ST6GALNAC5 were among the identified enzymes. The proxy approach to the study of glycosylation control by miRNAs has been recently reviewed in detail [17]. In this section, we will discuss how glycosyltransferase genes are regulated by ncRNAs. We have classified glycosyltransferases in the following groups: (i) initiating glycosyltransferases, which catalyze the initial steps of the biosynthesis of *N*- and *O*-glycans and glycolipids; (ii) core-extending glycosyltransferases, which elaborate core structures of *N*- and *O*-glycans and glycolipids; (iii) elongating glycosyltransferases, which elongate carbohydrate chains shared by different glycoconjugate classes; and (iv) capping glycosyltransferases, which cap carbohydrate chains shared by different glycoconjugate classes. In addition, we have considered two main classes of sugar-binding molecules, namely galectins and siglecs. A large part of the literature deals with cancer, but a relevant number of studies deal with the ncRNA regulation of glycosylation in other pathological contexts. The numerous examples of glycogene regulation by ncRNAs are summarized in Table 1.

### 4.1. Initiating Glycosyltransferases

#### 4.1.1. *N*-Linked Chains

The initial step of *N*-glycans biosynthesis consists of the addition of GlcNAc to dolichol phosphate and is mediated by DPAGT1 (Figure 2). In esophageal squamous cell carcinoma, DPAGT1 promotes growth and is down-regulated by miR-485-5p which, in turn, is sponged by lncRNA LINC00467. Consequently, the latter behaves as an oncogene [29]. A second example of regulation of an initiating *N*-glycosyltransferase is provided by the α3 mannosyltransferase ALG3, which is involved in a biosynthetic step of the GlcNAc2, Man9, Glc3 precursor (Figure 2). ALG3 contributes to the malignancy of non-small-cell lung cancer (NSCLC) and is negatively regulated by miR-98-5p [19].

#### 4.1.2. Mucin-Type *O*-Glycosylation

In bladder cancer, miR129 exerts growth inhibition by repressing GALNT1 [53] (Figure 3A). In ovarian cancer, lncRNA PSMA3-AS1 promotes cell proliferation, migration and invasion by sponging miR-378a-3p, which targets GALNT3. This leads to GALNT3 overexpression and PI3K/Akt pathway activation [58]. GALNT4 is one of the few GALNTs with preference for partially GalNAc-glycosylated substrates, modifying the available sites not employed by other GALNTs. In prostate cancer [59] and NSCLC [60], GALNT4 behaves as a tumor-promoting gene. In the former it is targeted by miR-506-3p, whereas in the latter it is targeted by miR-365b. By contrast, in hepatocellular carcinoma, GALNT4 behaves as a tumor-restraining enzyme, inhibited by miR-9 [61]. GALNT7 also prefers partially GalNAc-glycosylated substrates. Reports indicate opposite roles in different contexts. In fact, GALNT7 is tumor-promoting in cervical carcinoma (targeted by miR-30e [64] and miR-214 [65]), esophageal squamous cell cancer (targeted by miR-214 [67]), laryngeal carcinoma [66] and colorectal cancer [63], both targeted by miR-34a. On the contrary, in melanoma [62] and hepatocellular carcinoma [68] GALNT7 plays a tumor-restraining role. GALNT7 inhibits cytolytic activity of natural killer cells associated with lung cancer. Consistently, miR-30c which targets GALNT7 enhances NK cytotoxicity [69]. GALNT10 exerts a tumor-promoting activity in cholangiocarcinoma [55] and in hepatitis B virus-associated hepatocellular carcinoma [56], regulated by DLGAP1-AS2/miR -505 in the former and by miR-122 in the latter. miR-125a inhibits ovarian cancer proliferation and invasion by repressing GALNT14 expression [57].

The following are examples of mucin-type *O*-glycosylation regulation by ncRNA in non-neoplastic conditions. miRNA let-7i-5p exacerbates kidney fibrosis by targeting GALNT1 [54]. MiR-378 binds competitively to both the 3’ UTR of the nephronectin (an extracellular glycoprotein increasing osteoblast differentiation) mRNA and the GALNT7 transcript. Nephronectin glycosylation by GALNT7 creates a complex balance modulating osteoblast differentiation [70].

#### 4.1.3. *O*-Linked GlcNAc

In esophageal cancer, malignancy is increased by OGT over-expression due to down-regulation of miRNA-485-5p [85]. A similar condition was observed by miR-15a and miR-26a in clear-cell renal cell carcinoma [86]. In hepatocarcinoma, the regulation of RAF1 oncogene, which is involved in progression, offers a good example of the interplay between a glycosyltransferase, such as OGT, miRNAs and lncRNAs. In fact, OGT mediates RAF1 *O*-GlcNAcylation, promoting its stability. MiR-424-5p targets OGT but it is sponged by the lncRNA XIST (which plays a major role in the mechanisms of chromosome X inactivation in females) [87]. *O*-GlcNAcylation is involved in muscular homeostasis. In cancer, a decline in skeletal muscle mass is often observed. It has been shown that miR-122, encapsulated in extracellular vesicles and released by breast cancer cells, suppresses OGT, reducing *O*-GlcNAcylation of ryanodine receptor RYR1, resulting in skeletal muscle proteolysis [88]. Besides the many examples of glycosylation regulation by ncRNA, opposite cases also exist. In particular, the miR-483-3p production in liver cancer cells is made possible by the *O*-GlcNAcylation of the transcriptional complex at the miR-483 promoter [120]. Other examples of miRNA-mediated OGT regulation in non-neoplastic conditions are presented as follows. OGT targeting by miR-501-3p and miR-619-3p is a key factors in the regulation of hepatitis C virus assembly and infectivity [121]. Multiple sclerosis is a de-myelinating autoimmune disease in which the helper T cell subpopulation Th17 plays a major role. The transcription factor RORγt, which is the key determinant for Th17 differentiation, requires *O*-GlcNAcylation. OGT targeting by miRNA-15b suppresses Th17 differentiation, ameliorating demyelination in animal models of multiple sclerosis [89]. Apoptosis of cardiomyocytes, an event closely associated with congestive heart failure, is prevented by *O*-GlcNAcylation. Targeting of OGT by miR-423-5p promotes apoptosis in cardiomyocytes [90]. As shown in a recent review [17], OGT is a glycosyltransferase tightly regulated by miRNAs.

#### 4.1.4. Glycolipids

The number of studies reporting regulation by the ncRNA network of the first steps of glycolipid biosynthesis is surprisingly small. B4GALT5 synthesizes lactosylceramide, the core portion of glycolipids (Figure 4). Acute myeloid leukemia progression is promoted by B4GALT5 and circ0009910 which sponges miR-491-5p, activating the PI3K/AKT signaling pathway [24].

### 4.2. Core-Extending Glycosyltransferases

In this class are included the glycosyltransferases acting directly on the core structures of *N*- and *O*-linked chains of glycoproteins and of glycolipids. 

#### 4.2.1. *N*-Linked Chains

Core-extending glycosyltransferases of *N*-linked chains include fucosyltransferase 8 (FUT8), mediating the addition of α6-linked fucose to the innermost GlcNAc of the core, and GlcNAc transferases 1-5, product of genes MGAT1-MGAT5, which add GlcNAc to the Man residues of the trimannosyl core (Figure 2). 

FUT8: In cancer, FUT8 increase is unambiguously associated with malignancy. FUT8 is directly targeted by miR-122 and miR-34a in hepatocarcinoma [48], as well as by miRNA-198-5p in NSCLC [50], leading to reduced malignancy in both cases. In oral squamous cell carcinoma, FUT8 inhibitor miR-186 is sponged by lncRNA SNHG1 [51]. In breast cancer, miR-10b enhances FUT8 expression through noteworthy mechanisms, highlighting a more complex glycosyltransferase/miRNA relationship [122]. In fact, FUT8 transcription requires phosphorylated STAT3. In turn, the transcription factor activator protein 2γ (AP-2γ), which is targeted by miR-10b, binds to STAT3, preventing its phosphorylation. Thus, inhibition of AP-2γ by miR-10b results in FUT8 activation [122]. FUT8 transcription in liver cancer involves the indirect (through Hsp90 and MUC1) activation of the STAT3/JAK1 cascade, which is potentiated by antisense RNA HOTAIR [49]. Another interesting mechanism is the basis of FUT8 regulation by lncRNA LEF1-AS1 in colorectal cancer. This lncRNA recruits the histone methyltransferase MLL1 (product of the *KMT2A* gene) to the LEF1 promoter site, resulting in increased LEF1 expression and FUT8 transcription via the Wnt/β-catenin pathway [123]. FUT8-mediated core fucosylation of various profibrotic signals is a crucial event in the pathogenesis of renal interstitial fibrosis, a pathology secondary to chronic kidney diseases. FUT8 targeting by miR-34c-5p delivered by mesenchymal stem cells ameliorates the disease [52].

MGATs: MGAT5 is a well-known GlcNAc transferase involved in malignancy, particularly in metastasis formation in various systems [124]. In breast cancer, decreased miR-124-3p, which targets MAGAT5, promotes proliferation and metastasis [84]. On the other hand, in mammary cells, miR-424 has been shown to down-regulate the expression of MGAT4A, the GlcNAc transferase which adds GlcNAc in β4 linkage to the trimannosyl core (Figure 2). The presence of this modification promotes malignancy through cyclin D1 activation [83]. A complex mechanism of glycosylation regulation by ncRNAs is provided by LINC00173, which promotes Wilms’ tumor progression. LINC00173 stabilizes MGAT1 mRNA by recruiting the HNRPA2B1 ribonucleoprotein, resulting in mucin MUC3A *N*-glycosylation and tumor progression [81]. One of the hallmarks of Alzheimer’s disease is the presence of a hyperphosphorylated form of tau protein, which is the basis of neurofibrillary tangle formation. Tau pathology is attenuated by miRNA-23b through MGAT3 targeting [82].

#### 4.2.2. *O*-Linked Chains

Core 1 structures: The biosynthesis of core 1 *O*-linked structures starts with the addition of Gal to GalNAc by galactosyltransferase C1GALT1 (Figure 3), which requires the presence of the molecular chaperone COSMC. C1GALT1 expression promotes lung cancer progression by oncogene RAC1 up-regulation. This activity is negatively regulated by miR-181d-5p, which targets C1GALT1 [25]. C1GALT1 promotes malignancy even in bladder cancer, but it is inhibited by miR-1-3p, which is sponged by circHP1BP3 [26]. In aging colons, C1GALT1 expression is decreased, making mucus glycosylation defective and increasing susceptibility to colitis. This is partially due to overexpression of miR-124-3p, which targets C1GALT1 [27]. In IgA nephropathy, IgGA1 antibodies are aberrantly *O*-glycosylated because of the increased expression of miR-374b, which targets COSMC [28]. 

Core 2 and 4 structures: The β6 GlcNAc transferase GCNT3 is responsible for core 2 and core 4 *O*-glycan biosynthesis (Figure 3). Down-regulation by miR15b inhibits colon and pancreatic cancer growth [72]. MiR-BART1-5p is an Epstein–Barr virus (EBV)-encoded miRNA expressed in all stages of the viral infection. In EBV-associated gastric cancer, miR-BART1-5p targets GCNT3 to repress cell proliferation and migration [73]. In lung cancer, GCNT3 is up-regulated by LINC00511, which sponges its inhibitor miR-195-5p [74]. Noteworthy, GCNT3 appears to exert a tumor-restraining activity in colon and pancreatic cancer [72] and a tumor-promoting activity in gastric and lung cancers [73,74].

Core 3 structures: Core 3 glycans provide another example of miRNA regulation by glycosylation rather than the opposite. MUC1 is a heavily *O*-glycosylated membrane glycoprotein comprising an extracellular amino-terminal domain involved in cell adhesion and an intracellular C-terminal domain involved in cell signaling. In colon cancer, core 3 glycans synthesized by B3GNT6 decorate the *N*-terminal portion of MUC1, hindering the nuclear migration of the C-terminal portion of the protein. The absence of the C-terminal portion of MUC1 in the nucleus triggers the transcription of p53 and miR-200c, enhancing the mesenchymal to epithelial transition (which is the opposite of the more popular epithelial to mesenchymal transition) [125]. 

α2,6-Sialylation: ST6GALNAC transferases 1 and 2 mediate the α2,6-sialylation of the innermost GalNAc of *O*-linked chains. MiR-30d-5p is involved in NSCLC progression and is proposed to act by regulating several genes and their downstream pathways. ST6GALNAC1 is among these genes, indicating a possible role of mucin-type *O*-glycans [126]. MiR-182 and miR-135b increase malignancy of colorectal cancer cells by targeting ST6GALNAC2 which behaves as a tumor-restraining enzyme in this system [109,110].

#### 4.2.3. Glycolipids

The core-extending glycolipid sialyltransferase ST3GAL5 is targeted by miR-26a, miR-548l and miR-34a, resulting in inhibition of hepatocarcinoma progression [99].

### 4.3. Elongating Glycosyltransferases

**Figure 5:**
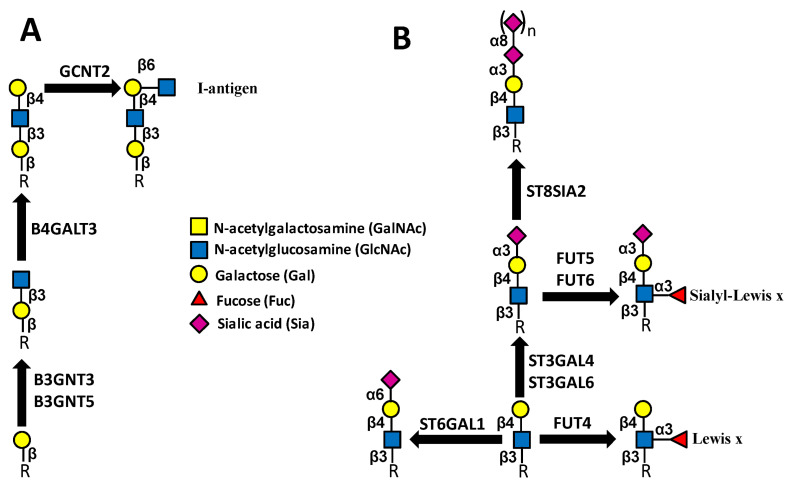
Elongating (**A**) and capping (**B**) glycosyltransferases. Enzymes catalyzing the same reactions but not mentioned in the text are not shown.

Elongating glycosyltransferases add sugars, such as GlcNAc and Gal, to core structures of *N*- and *O*-linked chains and glycolipids, forming linear or branched polylactosaminic structures (Figure 5A).

#### 4.3.1. GlcNAc Transferases

The enzyme B3GNT3, involved in polylactosamine biosynthesis, is targeted by miR-149-5p. In lung cancer, it promotes progression and is associated with poor prognosis [20]. B3GNT5 participates in elongation of glycolipids. LncRNA MIR44352HG is a well-recognized oncogene, regulating various signaling pathways. In liver cancer, it promotes progression by sponging miR1365p, leading to B3GNT5 upregulation [21]. GCNT2, a β6 GlcNAc transferase crucial for the biosynthesis of I antigen (Figure 5) whose expression is positively associated with malignancy in colon cancer cell lines, is targeted by miR-199a/b-5p [71].

#### 4.3.2. Gal Transferases

B4GALT3 adds Gal to GlcNAc, forming type 2 lactosaminic chains. In human cervical cancer cells, B4GALT3 is unconventionally up-regulated by miR-27, contributing to oncogenic activity through stabilization of β1 integrins [22]. LncRNA DANCR inhibits cell differentiation often associated with cancer. In neuroblastoma, DANCR promotes B4GALT3 expression and malignancy by sponging miR-338-3p [23].

### 4.4. Capping Glycosyltransferases

Capping glycosyltransferases add terminal sugars to extended carbohydrate chains. This group includes mainly, but not exclusively, fucosyltransferases and sialyltransferases. Among the most relevant terminal-fucosylated structures are the Lewis^x^ (Le^x^) and its sialylated counterpart sialyl Lewis^x^ (sLe^x^) (Figure 5). The overexpression of these structures in several cancers correlates with malignancy through different mechanisms [127], including binding to cells’ adhesion molecules of the selectin family [128,129]. While the biosynthesis of Le^x^ occurs through the simple addition of a α1,3-linked Fuc on a type 2 chain, the biosynthesis of sLe^x^ requires the preliminary addition to the type 2 chains of a α2,3-linked Sia (Figure 5)

#### 4.4.1. Fucosyltransferases

FUT4. FUT4 is mainly responsible for the biosynthesis of Le^x^, while its contribution to the biosynthesis of sLe^x^ is considered marginal [130]. Numerous studies report FUT4 regulation by ncRNAs in various cancers and its contribution to malignancy. In colorectal cancer, FUT4 is down-regulated by miR-200c [30] and by miR-26a/26b [31]. The latter is sponged by lncRNA MALAT1, delivered through exosomes [32]. Additionally, in breast cancer, FUT4 and its associated glycans exert a cancer-promoting activity, which is limited by miR-224-3p [33], miR-200b/c [36,37] and miR-493-5p [34], which is sponged by lncRNA GAS6-AS2 [35]. In bladder cancer, FUT4 is controlled by miR-371b-5p, which is sponged by lncRNA AC114812.8 [38]. Among hematological cancers, FUT4 has been shown to increase malignancy of leukemia stem cells due to miR-29b, which inhibits the transcription factor Sp1 binding to FUT4 promoter [39]. In multiple myeloma, lncRNA HOXB-AS1 promotes growth. ELAVL1 is a member of the ELAVL family of RNA-binding proteins whose role is to stabilize mRNAs by binding to their 3′UTR. The tumor-promoting activity of HOXB-AS1 in myeloma is partly due to its ability to promote ELAVL1 binding to FUT4 mRNA, resulting in its stabilization [40]. Medulloblastoma cancer stem cells are positive for CD133, as well as for sLe^x^ (CD15). MiR199b-5p has been reported to target FUT4 in these cells [41] even if FUT4 is a poor sLe^x^ synthase. In a non-neoplastic context, FUT4 targeting by miR-26a/b [42] and miR-200b [43] reduces articular inflammation and uterine receptivity [44].

FUT5 and FUT6: MiR-125a-3p can reduce malignancy of colorectal cancer cells by targeting FUT5 and FUT6 and the downstream PI3K-AKT pathway [45]. Additionally, lncRNA HOTAIR supports colorectal cancer progression by interacting with miR-326 and FUT6, increasing CD44 fucosylation and stimulating the PI3K/AKT/mTOR pathway [46]. On the other hand, in breast cancer, FUT6 appears to play a tumor-restraining role. In fact, FUT6 inhibition by miRNA-106b promotes cell migration, invasion and proliferation [47].

#### 4.4.2. Sialyltransferases

We will distinguish between ST3GAL—which catalyzes the addition of Sia in α2,3 linkage to Gal—ST6GAL and ST6GALNAC—which mediate the addition of Sia in α2,6-linkage to Gal or GalNAc, respectively—and polysialyltransferases, which add Sia in α2,8 linkage to an underlying Sia residue.

ST3GAL: ST3GAL1 is the major sialyltransferase catalyzing the sialylation of core 1 *O*-linked chains, leading to sialyl-T formation (Figure 3). The lncRNA MEG3, expressed only by the maternally inherited chromosome, shows tumor-suppressor activity. In renal cell carcinoma, it regulates binding of the transcription factor JUN to the ST3GAL1 promoter, reducing its transcription and leading to reduced EGFR sialylation, increased phosphorylation and activation of the PI3/AKT pathway [93]. ST3GAL2 is a crucial sialyltransferase acting on *O*-linked chains and glycolipids (Figure 3 and Figure 4). The intestinal bacterial pathogen *Campylobacter jejuni* empowers its infectivity, inducing glycosylation changes. One mechanism involves the inhibition of ST3GAL2 by miR-615-3p [94]. Sialylation of a Galβ1,4GlcNAc unit, operated by ST3GAL4 or ST3GAL6, is a crucial step in the biosynthesis of sLe^x^ antigen, which is followed by subsequent α1,3-fucosylation (Figure 5). In kidney cancer, ST3GAL4 is targeted by miR-193a-3p and miR-224 and seems to play a tumor-restraining role through the PI3K/AKT pathway [95]. By contrast, in chronic myeloid leukemia cells, ST3GAL4 up-regulation resulting from the downregulation of their inhibitors miR-224 and let-7i contributes to cell survival and chemoresistance [96]. In colon carcinoma cells, ST3GAL4 targeting by miR-370 inhibits P-selectin-induced cell adhesion by targeting ST3GAL4 [97]. Modulation of ST3GAL4 by miR-193b also plays a role in inflammatory disease, such as osteoarthritis, by regulating CD44 sialylation through the NF-kB pathway [98]. In hepatocarcinoma, ST3GAL6 promotes malignancy and is targeted by miR-26a [100], while in lung cancer it reduces malignancy, acting on EGFR signaling [101]. Besides coding transcript(s), the *ST3GAL6* gene also generates antisense transcript ST3GAL6-AS1, derived from the promoter region and circRNA. In lung cancer, ST3GAL6-AS1 expression parallels that of ST3GAL6, restraining malignancy [101]. In colorectal cancer, ST3GAL6-AS1 exerts a tumor-restraining activity by recruiting histone methyltransferase MLL1 to the ST3GAL6 promoter, resulting in increased *ST3GAL6* transcription and α2,3-sialylation and PI3K/AKT inhibition [102]. On the other hand, in multiple myeloma, ST3GAL6-AS1 promotes invasion [131] by increasing ST3GAL6 expression. This was obtained through ST3GAL6-AS1-mediated inhibition of the heterogeneous nuclear ribonucleoprotein A2B1 (*HNRNPA2B1* gene), a protein which stabilizes the ST3GAL6 transcript [103].

ST6GAL and ST6GALNAC: α2,6sialyltransferases include ST6GAL1 and 2, which mediate the addition of α2,6-linked Sia to Gal and ST6GALNAC transferases which add sialic acid to GalNAc. ST6GAL1 is by far the major ST6GAL. In liver cancer, ST6GAL1 stimulates progression, and its expression is regulated by miR-9 [104] and by miR-195-3pc/lncRNA TINCR [105]. ST6GAL1 also affects the exosomal release of a broad range of miRNA. In fact, the activity of neutral sphingomyelinase-2, a key enzyme in exosomal sorting of miRNA, is regulated by α2,6-sialylation. Consequently, differential expression of ST6GAL1 underlies differential miRNA sorting [132]. In T-cell acute lymphoblastic leukemia, high ST6GAL1 is associated with drug resistance. It is regulated by miR-150, which is sponged by ZF-AS1 and modulates sialylation of EGFR via the PI3K/Akt pathway [107]. On the other hand, in triple-negative breast cancer, ST6GAL1 exerts a tumor-restraining activity. In fact, up-regulation of miR-214-3p, which targets ST6GAL1, is associated with malignancy [106]. ST6GAL1 has been shown to be regulated by miR199a, affecting sialylation of nectin-like molecule 2 and increasing ErbB2/ErbB3 signaling [108]. An in silico study in alcohol-related esophageal cancer has identified some lncRNA/miRNA interactions potentially regulating ST6GAL1 [133]. Both ST6GALNAC4 and ST6GALNAC5 are mainly involved in the biosynthesis of the GD1α ganglioside. In human follicular thyroid carcinoma, ST6GALNAC4, which is inhibited by miR-4299, promotes malignancy [111]. On the other hand, in prostate cancer, ST6GALNAC5, which is targeted by miR182, exerts a tumor-restraining role [112].

Polysialyltransferases. Several ST8SIA, including ST8SIA1 (GD2 synthase), mount a single Sia unit on Sia, generating the Siaα2,8Sia disaccharide. However, only two members of the ST8SIA family, namely ST8SIA2 and ST8SIA4, can synthesize long linear chains of polysialic acid, such as those present on the neural cell adhesion molecule (NCAM) and a few other glycoproteins. ST8SIA1 is generally associated with malignancy [134]. In colorectal cancer progression, it is inhibited by miRNA-33a and let-7e ST8SIA1 [113], while in prostate cancer progression, it is stimulated by the lncRNA MIR44352HG, resulting in FAK/AKT/β-catenin signaling pathway activation [114]. In ischemia/reperfusion brain models, ST8SIA2 is increased. This change is mediated by increased expression of the lncRNA TUG1, which sponges miR-3072-3p targeting ST8SIA2 [115]. The miR-26a-b/MALAT1 axis already described for FUT4 regulation in colorectal cancer also modulates ST8SIA4 in breast cancer cell lines [116,117]. ST8SIA4, targeted by miR-144-5p and miR-451a, also promotes growth in cholangiocarcinoma cells [119]. On the other hand, in follicular thyroid carcinoma, ST8SIA4, targeted by miR-146a and miR-146b, inhibits proliferation, migration and invasion [118]. 

#### 4.4.3. AB0 Glycosyltransferases

Three allelic forms of a single genetic locus regulate the biosynthesis of the AB0 antigens. The allele responsible for the “A” blood group encodes a α1,3GalNAc transferase; the one responsible for the “B” group encodes a highly homologous α1,3 Gal transferase; the “0” antigen results from a null allele. In rare cases, the weak expression of the A/B antigens cannot be explained by genetic variations in the glycosyltransferase coding region. Even the disappearance of AB0 antigens during carcinogenesis is not fully explained. A possible explanation is provided by the observation that miR-331-3p and miR-1908-5p directly target the mRNA of glycosyltransferases A and B [18].

## 5. Regulation of Sugar-Binding Molecules by ncRNAs

### 5.1. Galectins

Galectins are epigenetically regulated [135] soluble galactose-binding molecules, which exert an extremely wide array of biological functions [136]. In cancer, some galectins exert tumor-promoting activity, while others play the opposite role. Galectin-3, a product of the *LGALS3* gene, is frequently associated with malignancy. In ovarian [75] and colorectal [76] cancer, it is targeted by miR-424-3p and miR-128, respectively. Growth of pancreatic cancer cells is inhibited by miRNA-128-3p, delivered by exosomes released from human umbilical cord mesenchymal stem cells. In addition, the apoptosis of the nucleus pulposus cells (cells of the intervertebral disc) induced by galectin-3 is inhibited by miR-299-5p, which is in turn sponged by circRNA RERE [78]. Galectin-9, a product of the *LGALS9* gene, exerts a tumor-promoting activity in liver cancer, targeted by miR-22 [80], and a tumor-restraining activity in colon cancer [79] in which it is targeted by miR-455-5p.

### 5.2. Siglecs

Siglecs are sialic acid receptors of the immunoglobulin family expressed mainly by cells of the immune system, playing a fundamentally inhibitory role and aiding tumors in escaping immune recognition [137]. SIGLEC15 behaves as a tumor immune suppressor. In clear-cell renal cell carcinoma, LINC00973 sponges miR-7109-3p, resulting in increased SIGLEC15 expression [91]. Analogously, in hepatocellular carcinoma, SIGLEC15 is targeted by miR-582-5p, which is sponged by lncRNA TUG1 [92].

## 6. Non-Coding RNAs Derived from Glycosyltransferase Genes but Not Involved in Glycogene Regulation

A number of ncRNAs derived from glycosyltransferase genes do not modulate glycogenes but exert a function on other basic cellular mechanisms. MGAT3-AS1 is an lncRNA derived from the antisense transcription of an intronic sequence of the MGAT3 gene. Low levels of this transcript correlate with delayed rejection [138] but an increased risk for viremia of polyomavirus and cytomegalovirus after kidney transplantation [139]. 

Several antisense glycosyltransferase lncRNAs affect cancer cell malignancy. B3GALT5-AS1 contributes to the progression of gastric cancer by up-regulating the expression of the α1 subunit of casein kinase Ii (product of the CSNK2A1 gene), which is involved in a variety of signaling pathways [140]. ST8SIA6-AS1 lncRNA (also known as APAL) is overexpressed and associated with poor prognosis in a variety of cancers [141]. Its silencing causes mitotic catastrophe and massive apoptosis in human cancer cells [141]. ST8SIA6-AS1 increases malignancy by regulating miR-142-3p [142], miR-338-3p [143] and miR-651-5p [144] in hepatocellular carcinoma cells, while in triple-negative breast cancer it drives cell proliferation and metastasis by targeting miR-145-5p, resulting in p53 pathway inactivation [145].

Even circRNAs contribute to regulate cancer cell growth. CircRNA ST3GAL6 displays a tumor-restraining activity in gastric cancer through autophagy set by the FOXP2/MET/mTOR axis [146]. In bladder cancer cells, circRNA ST6GALNAC6 behaves as a tumor-suppressor by increasing the sensitivity to ferroptosis, a type of programmed cell death induced by iron accumulation [147]. Finally, circRNA FUT10 sponges miR-365a-3p, inhibiting its binding with homeobox A9. These mechanisms regulate the regenerative potential of aged skeletal muscle stem cells [148].

## 7. Common Patterns of Glycogene Modulation by ncRNA in Cancers

**Figure 6:**
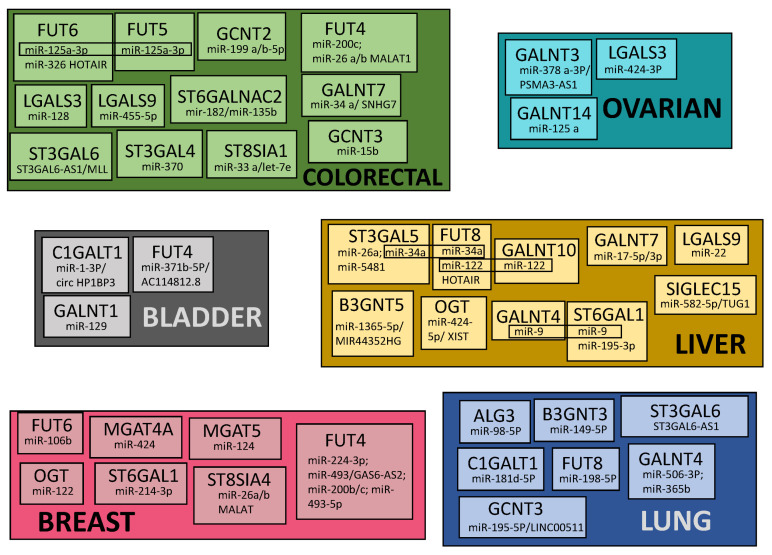
Glycogenes modulated by ncRNAs in different cancer types. Only cancers with at least 3 modulated glycogenes are reported. MiRNAs modulating different enzymes in the same cancer type are boxed.

In Figure 6 are shown the glycogenes modulated by ncRNAs in different cancer types (only cancers with at least three modulated glycogenes are reported). These data, together with those reported in Table 1, show that only a few molecules undergo common modulation by ncRNAs in different cancers. In particular, FUT4 displays common modulation in breast and colon cancers by miR-200c. On the other hand, some miRNAs modulate different enzymes of the same malignancy. This is the case of liver cancer, in which miR-9 modulates GALNT4 and ST6GAL1, miR-122 modulates FUT8 and GALNT10 and miR-34 modulates ST3GAL5 and FUT8. In addition, miR-125a-3p modulates FUT5 and FUT6 in colorectal cancer. Together, these data are consistent with the existence of a very intricate and fragmented network of glycosylation regulation by ncRNAs.

## 8. Conclusions

Although many examples of glycosylation control by the ncRNA network have been published in recent years, they probably represent just the tip of the iceberg. It is reasonable to hypothesize that nearly all the components of the glycosylation machinery undergo regulation by ncRNAs because both kinds of molecules concur to define precisely the amount of protein molecules and their biological function. These mechanisms are crucial to the health of highly complex multicellular organisms, such as mammals. However, some glycogenes appear to be more frequently regulated by ncRNAs than others, suggesting that they require a particularly precise regulation. According to a recent hypothesis, deregulation of these genes is associated with complex diseases, such as such cancer and inflammatory conditions [17,149]. The recently discovered glyco-RNAs [5] are sialylated and located on the plasma membrane and found to be able to interact with siglecs. The intrinsic nature of the technique used for glyco-RNA isolation, which is based on a sialic acid analogue, limits—for the moment—the study to sialylated glyco-RNAs, but it is likely that neutral glyco-RNAs will be discovered in the future. The existence of this new kind of glycoconjugate establishes a new paradigm in glycobiology. Its impact on human health is, at the moment, unpredictable. Unlike other small RNAs, which are intracellular, glyco-RNA are exposed on the cell membrane in close contact with the immune system. On this basis, their possible involvement in auto-immune diseases has been proposed [5]. The emerging picture of the mutual relationship between ncRNA and glycosylation paves the way for conceptually new therapies. 

## Figures and Tables

**Table 1:** Glycogenes modulated by ncRNAs.

Target Glycogene	Upstream ncRNA	Downstream ncRNA	Downstream Target	Tissue and Reference
A/B glycosyltransferases	miR-331-3p miR-1908-5p			Biosynthesis of AB0 antigens [18]
ALG3	miR-98-5p			Lung cancer [19]
B3GNT3	miR-149-5p			Lung cancer [20]
B3GNT5	miR1365-5p	MIR44352HG		Liver cancer [21]
B4GALT3	miR-27		β1 integrins	Cervical cancer [22]
	miR-338-3p	DANCR		Neuroblastoma [23]
B4GALT5	miR-491-5p	circ_0009910	miR-491-5p	Acute myeloid leukemia [24]
C1GALT1	miR-181d-5p		RAC1	Lung cancer [25]
	miR-1-3p	circ HP1BP3		Bladder cancer [26]
	miR-124-3p			Aging colon [27]
COSMC	miR-374b			IgA nephropathy [28]
DPAGT1	miR-485-5p	LINC00467		Esophageal cancer [29]
FUT4	miR-200c			Colorectal cancer [30]
	miR-26a/b	MALAT1	PI3/AKT	Colorectal cancer [31,32]
	miR-224-3p			Breast cancer [33]
	miR-493	GAS6-AS2		Breast cancer [34,35]
	miR-200c			Breast cancer [36]
	miR-493-5p			Breast cancer [34]
	miR-200b			Breast cancer [37]
	miR-371b-5p	AC114812.8		Bladder cancer [38]
	miR-29b	Sp1	CD44	Leukemia stem cells [39]
		HOXB-AS1		Multiple myeloma [40]
	miR-199b-5p			Medulloblastoma [41]
	miR26a/b		NFkB	Osteoarthritis [42]
	miR200b			Arthritis [43]
	miR200c			Uterine receptivity [44]
FUT5	miR-125a-3p		PI3K/AKT	Colorectal cancer [45]
FUT6	miR-125a-3p		PI3K/AKT	Colorectal cancer [45]
	miR-326	HOTAIR	CD44/PI3K/AKT	Colorectal cancer [46]
	miR-106b			Breast cancer [47]
FUT8	miR-34a, miR-122			Liver cancer [48]
		HOTAIR	JAK/STAT3	Liver cancer [49]
	miR-198-5p			Lung cancer [50]
	miR-186	SNHG1	MMP2/MMP9	Oral cancer [51]
	miR-34c-5p			Renal interstitial fibrosis [52]
GALNT1	miR-129			Bladder cancer [53]
	let-7i-5p			Kidney fibrosis [54]
GALNT10	mir-505	DLGAP1-AS2		Cholangiocarcinoma [55]
	miR-122			Liver cancer [56]
GALNT14	miR-125a		MMP2 and MMP9	Ovarian cancer [57]
GALNT3	miR-378a-3p	PSMA3-AS1	PI3K/Akt	Ovarian cancer [58]
GALNT4	miR-506-3p			Lung cancer [59]
	miR-365b			Lung cancer [60]
	miR-9			Liver cancer [61]
GALNT7	miR-30b/30d			Melanoma [62]
	miR-34a	SNHG7	PI3K/Akt/mTOR	Colorectal cancer [63]
	miR-30e			Cervical cancer [64]
	miR-214			Cervical cancer [65]
	miR-34a/c			Laryngeal cancer [66]
	miR-214			Esophageal cancer [67]
	miR-17-5p/miR-17-3p			Liver cancer [68]
	miR-30c		PI3K/AKT	Natural killer activity [69] in lung cancer
	miR-378			Osteoblast differentiation [70]
GCNT2	miR-199a/b-5p			Colorectal cancer [71]
GCNT3	miR-15b			Pancreatic and colorectal cancer [72]
	miR-BART1-5p			EBV-induced gastric cancer [73]
	miR-195-5p	LINC00511		Lung cancer [74]
LGALS3	miR-424-3p			Ovarian cancer [75]
	miR-128			Colorectal cancer [76]
	miR-128-3p			Pancreatic cancer [77]
	miR-299-5p	circRERE		Apoptosis of nucleus polposum cells [78]
LGALS9	miR-455-5p			Colorectal cancer [79]
	miR-22			Liver cancer [80]
MGAT1		LINC00173	mucin 3A	Wilms’ tumor [81]
MGAT3	miR-23b		Tau protein	Alzheimer’s disease [82]
MGAT4A	miR-424		cyclin D1	Breast cancer [83]
MGAT5	miR-124			Breast cancer [84]
OGT	miR-485-5p			Esophageal cancer [85]
	miR-15a/miR-26a			Kidney cancer [86]
	miR-424-5p	XIST	Raf1	Liver cancer [87]
	miR-122		RYR1	Breast cancer [88]
	miR-15b		RORγt	Th17 differentiation [89]
	miR-423-5p			Apoptosis of cardiomyocytes [90]
SIGLEC15	miR-7109-3p	LINC00973		Kidney cancer [91]
	miR-582-5p	TUG1		Liver cancer [92]
ST3GAL1		MEG3	EGFR/PI3K/AKT	Kidney cancer [93]
ST3GAL2				Gut infection [94]
ST3GAL4	miR-193a-3p miR-224		PI3K/AKT	Kidney cancer [95]
	miR-224, let-7i			Chronic myeloid leukemia [96]
	miR-370			Colorectal cancer [97]
	miR193-b		CD44/NF-kB	Arthritis [98]
ST3GAL5	miR-26a, miR-548l, miR-34a			Liver cancer [99]
ST3GAL6	miR-26a		AKT/mTOR	Liver cancer [100]
	ST3GAL6-AS1		EGFR	Lung cancer [101]
	ST3GAL6-AS1	MLL1	ST3GAL6	Colorectal cancer [102]
	ST3GAL6-AS1	HNRNPA2B1	ST3GAL6	Multiple myeloma [103]
ST6GAL1	miR-9		β1-integrins/FAK	Liver cancer [104]
	miR-195-3p	TINCR	NFkB	Liver cancer [105]
	miR-214-3p			Breast cancer [106]
	miR-150	ZF-AS1	EGFR/PI3K/Akt	Acute lymphoblastic leukemia [107]
	miR-199a		ErbB2/ErbB3	Various cancers [108]
ST6GALNAC2	miR-182/miR-135b		PI3K/AKT	Colorectal cancer [109,110]
ST6GALNAC4	miR-4299			Thyroid cancer [111]
ST6GALNAC5	miR-182			Prostate cancer [112]
ST8SIA1	miR-33a/let-7e			Colorectal cancer [113]
		MIR44352HG	FAK/AKT/β-catenin	Prostate cancer [114]
ST8SIA2	miR-3072-3p	TUG1		Brain ischemia [115]
ST8SIA4	miR-26a/b	MALAT1		Breast cancer [116,117]
	miR-146a/b		PI3K-AKT-mTOR	Thyroid cancer [118]
	miR-144-5p/miR-451a			Cholangiocarcinoma [119]

## Data Availability

Not applicable.

## Abbreviations

APAL	aurora A/polo kinase 1
AP-2γ	activator protein 2γ
circRNA	circular RNA
DANCR	differentiation antagonizing non-protein-coding RNA
DLGAP1	discs large-homolog-associated protein 1
DPAGT1	dolichyl-phosphate *N*-acetylglucosaminephosphotransferase 1
EMT	epithelial to mesenchymal transition
EBV	Epstein–Barr virus
HNRPA2B1	Heterogeneous Nuclear Ribonucleoprotein A2/B1
HOTAIR	HOX transcript antisense RNA
LEF1	Lymphoid enhancer binding factor 1
Le^x^	Lewis^x^
lncRNA	long non-coding RNA
MALAT1	metastasis-associated lung adenocarcinoma 1
miRNA	micro RNA
MUC1	mucin 1
NCAM	neural cell adhesion molecule
ncRNA	non-coding RNA
NSCLC	non-small-cell lung cancer
OGT	*O*-GlcNActransferase
PSMA3	proteasome 20S subunit alpha 3
RER	rough endoplasmic reticulum
RISC	RNA-induced silencing complex
RYR1	ryanodine receptor 1
sLe^x^	sialyl Lewis^x^
SNHG1	small nuclear RNA host gene 1
TINCR	TINCR ubiquitin domain containing
TUG1	Taurine up-regulated 1
XIST	X-inactivated specific transcript
